# Performance of Austenitic High-Nitrogen Steels under Gross Slip Fretting Corrosion in Bovine Serum

**DOI:** 10.3390/jfb15040110

**Published:** 2024-04-18

**Authors:** Alfons Fischer, Philipe Telouk, Christian Beckmann, Saskia Heermant, Adrian Wittrock, Jörg Debus, Markus A. Wimmer

**Affiliations:** 1 Max Planck Institute for Sustainable Materials, Microstructure Physics and Alloy Design, 40237 Duesseldorf, Germany; 2 Department of Orthopedic Surgery, Rush University Medical Center, Chicago, IL 60612, USA; markus_a_wimmer@rush.edu; 3 Laboratoire de Géologie, Université de Lyon, 69342 Lyon, France; telouk@ens-lyon.fr; 4 Department of Physics, TU Dortmund University, 44227 Dortmund, Germany; christian.beckmann@tu-dortmund.de (C.B.); saskia.heermant@tu-dortmund.de (S.H.); adrian.wittrock@tu-dortmund.de (A.W.); joerg.debus@tu-dortmund.de (J.D.)

**Keywords:** austenitic high-nitrogen steels, fretting corrosion, gross slip, ultra-mild wear, wear mechanisms

## Abstract

Modular artificial hip joints are a clinical standard today. However, the release of wear products from the head–taper interface, which includes wear particles in the nm size range, as well as metal ions, have raised concerns. Depending on the loading of such taper joints, a wide variety of different mechanisms have been found by retrieval analyses. From these, this paper concentrates on analyzing the contribution of gross slip fretting corrosion at ultra-mild wear rates using a bovine calf serum solution (BCS) as the lubricant. The parameters were chosen based on biomechanical considerations, producing wear rates of some ng/m wear path. In parallel, the evolution of tribomaterial (third bodies) was analyzed as to its constituents and generation rates. It has already been shown earlier that, by an advantageous combination of wear mechanisms and submechanisms, certain constituents of the tribomaterial remain inside the contact area and act like extreme-pressure lubricant additives. For the known wear and corrosion resistance of austenitic high-nitrogen steels (AHNSs), which outperform CoCrMo alloys even under inflammatory conditions, we hypothesized that such steels will generate ultra-mild wear rates under gross slip fretting. While testing AHNSs against commercially available biomedical-grade materials of CoCrMo and TiAlV alloys, as well as zirconia-toughened alumina (ZTA) and against itself, it was found that AHNSs in combination with a Ti6Al4V alloy generated the smallest wear rate under gross slip fretting corrosion. This paper then discusses the wear behavior on the basis of ex situ analyses of the worn surfaces as to the acting wear mechanisms and submechanisms, as well as to the tribological reaction products.

## 1. Introduction

In the US, about 1.5 million patients have to undergo hip and knee replacements, and these numbers are expected to increase in the future [1]. A total of 10% are revision operations due to infection, instability, aseptic loosening, and trunnionosis [2,3,4], while the latter is related to the release of wear products from the head–stem taper connection [5,6,7,8,9,10,11,12,13]. Such clinical problems have been of concern since the 1970s [14,15] and, today, affect nearly 40,000 patients annually in the US alone.

On the basis of retrieval studies [16,17], research about gross slip fretting corrosion with Ti6Al4V/CoCr29Mo6 couples was started, and has since gained the acting wear mechanisms in relation to the microstructures of the materials in contact [18]. It was shown that, besides the body, counterbody, lubricant, and environment, there are so-called third bodies or tribomaterials within the sliding interfaces. The third bodies are generated by the system and caught between the sliding bodies, thus influencing their wear behavior. On the basis of the fundamental work of Godet et al. [19,20,21], Rigney et al. [22,23], and, recently, Greiner et al. [24,25,26], it became clear that this nanostructured metallic–organic–ceramic composite with constituents from lubricant and wear products allowed for the extreme shear rates by the rotation of nano-grains or clusters of them [27,28]. No distinct differentiation is possible ex situ for the different layers of the tribomaterials, which are often arranged in chaotic eddies [22]. For the alteration of the chemical composition, the process belongs to the group of tribochemical reactions (TCRs) and is—by its predominantly mechanical nature—subsumed under the submechanism “mechanical mixing” [28,29]. This term had to be separated from “mechanical alloying” because in the tribological context it represents the generation of a nanostructured metallic–ceramic–organic composite with constituents from all elements of the tribosystem, as well as their tribological reaction products.

For the fretting corrosion of CoCr29Mo6/Ti6Al4V couples at ultra-mild wear rates, it was found that such a mechanically mixed layer was initiated by the microploughing of a submechanism of abrasion [30] (AB), but corroded away immediately on the CoCrMo side of the contact [18,31]. (It should be mentioned here that most of the published wear rates are wear rates in the rage of 10^−5^ mm^3^/Nm (or the m depth of the wear groove divided by the length of the wear path in m/m). In real applications, this would describe catastrophic wear behavior. Even high wear rates in tooling would be smaller than 10^−6^ (≈µm/m; mild wear), while those for mechanical engineering parts must be smaller than 10^−8^ (sub-mild wear). Thus, only ultra-mild wear rates of 10^−9^ m/m or mm^3^/Nm (e.g., as it would be for piston rings or artificial hip joints) and below from laboratory experiments would allow for analyses and an understanding of the characteristic mechanisms and submechanisms of long-lasting parts and devices.) Now, all such submechanisms that are based on either the monotonic (microploughing, the microcutting of abrasion, as well as the microwelding of adhesion (AD)) or cyclic (delamination, the indentation of surface fatigue (SF), as well as mechanical mixing) accumulation of plastic deformation require a certain amount of frictional work. Additionally, they trigger tribo-oxidation and tribocorrosion [32], while, in combination, all might generate wear products that add to the tribomaterial. Still, the term ‘generated’ does not automatically mean that they will be ejected from the system and become material loss [21]. Thus, the generated tribomaterial that remained within the wear grooves was analyzed separately from the ejected metal ions within the serum. This brought about differences in the orders of magnitude between the matter generated and the one ejected [18,33,34]. This underlines the load-carrying capacity of the tribomaterial (or third bodies) within the interface [18,33,35] and emphasizes its protective nature, which was already proposed in the 1980s [36]. This aspect might even be more important the more inflammatory the conditions are. It was also found that a rougher surface on one side of the contact leads to less wear over time [37], which could be mainly attributed to the shorter dwell time of the partly grainy tribomaterial within the real contact area [38]. Still, a boundary lubricating effect could be maintained by some of the wear products.

With a ceramic counterface—e.g., of zirconia-toughened alumina (ZTA) run against Ti6Al4V—these mechanisms and submechanisms change markedly, while there could be additional material transfer pointing towards microwelding from the metal to the ceramic side in vivo [39] and in vitro [34]. This was attributed to the higher friction values that have been found for metal–ceramic contacts in contrast to metal–metal ones [34,40]. Nevertheless, the release of metal ions was markedly reduced [34,41,42].

So far, it has become clear that, under fretting corrosion, most submechanisms contribute to the generation of wear products by forming tribomaterials. But tribocorrosion, which is the release of metal ions from mechanically depassivated surfaces, appears to be the most prominent submechanism for the ejection of wear products from the system.

Now, there are some serious concerns about the detrimental effects of Co ions and particles on human tissue [43,44,45,46]. In earlier studies, it has been shown by both in vitro and in vivo studies that Co- and Ni-free austenitic high-nitrogen steels (AHNSs) have adequate mechanical and chemical properties for biotribological applications and are biocompatible [47,48,49,50,51,52,53]. In particular, it was found that these steels have a better repassivation behavior than CoCrMo in electro-impedance spectroscopy experiments, as well as under reciprocating sliding wear in contact with Al_2_O_3_ [54]. Thus, AHNSs could, in principle, replace CoCrMo in some applications. Since tribocorrosion is the most prominent submechanism for the wear loss, we hypothesize that AHNSs should outperform CoCrMo alloys under fretting corrosion conditions as well. But, the Co- and Ni-free AHNSs—although not a new group of steels—have never been rigorously tested under gross slip fretting corrosion conditions, nor in comparison to other metallic and ceramic biomaterials.

Hence, in this study, fretting corrosion experiments with AHNSs in contact with Ti4Al6V, low-carbon CoCr29Mo6, ZTA ceramics, as well as with itself were run in order to relate the characteristic mechanisms and submechanisms to the tribological behavior. Afterwards, the results were compared to those of the metal–metal and metal–ceramic contacts, which were investigated earlier under the same tribological conditions, allowing for ultra-mild wear rates.

## 2. Materials and Methods

### 2.1. Materials

All materials were commercially available. The chemical compositions of the alloys are given in Table 1 according to the manufacturers’ certificates. The ZTA ceramic (Biolox Delta©, Ceramtec AG, Plochingen, Germany) consists of alumina, zirconia, and additives [34,55]. The solution heat treatments of the alloys were as follows:

- CoCr29Mo6C0.06—30 min/1050 °C/H_2_O [20];
- FeCr18Mn14Mo3CN0.9—45 min/1150 °C/H_2_O;
- FeCr18Mn13Mo3CN0.6—45 min/1145 °C/H_2_O.

**Figure 1:**
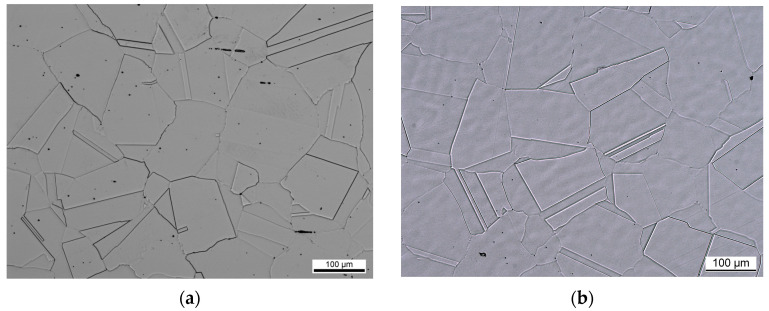
Microstructures of the investigated austenitic high-nitrogen steels. (**a**) FeCN0.9; (**b**) FeCN0.6.

The Ti alloy was used as received. The Vickers hardness was measured according to DIN EN ISO 6507-1. The microstructures showed typical appearances, as depicted in Figure 1.

Those of the other materials have already been published earlier [18,34]. The different materials will be designated by their shorter abbreviations, which are based on the base metals Fe and Co and the content of the substitutional Cr, Mn, and Mo, and the interstitial C and N alloying elements, as follows:

- CoCr29Mo6C0.06—CoC0.06;
- FeCr18Mn14Mo3CN0.9—FeCN0.9;
- FeCr18Mn13Mo3CN0.6—FeCN0.6.

The Ti alloy will be named as standardized Ti6Al4V [20,33] and the ceramic as ZTA.

The contact surfaces of the pins (diameter 12 ± 0.01 mm, height 7.1 ± 0.02 mm) were mechanically polished in various steps down to a 1 µm diamond suspension. The characteristics of the initial topographies were measured by a laser microscope at 20-fold (fluted) and 50-fold (polished) magnification (Keyence VK-x3000, Keyence Deutschland GmbH, Neu-Isenburg, Germany). The surfaces of the pins became slightly convex during polishing, with radii ranging between 3 and 8 m. The fluted (FL) cylinders were machined to a diameter of 13 ± 0.01 mm with a topography of circumferential ridges [38]. This led to a peak-like characteristic, with a positive R_sk_ and an R_ku_ being smaller than 3. The fluted topographies differed as to the depth of the valleys, as can be seen by the Ra in Table 2. Still, the distance between the ridges was the same for all samples at about 190 µm (R_sm_ = 190.3 ± 0.21 µm). The fluted topography did not resemble any known and commercially available taper topography.

### 2.2. Methods

#### 2.2.1. Fretting Test Rig Set-Up and Parameters

**Figure 2:**
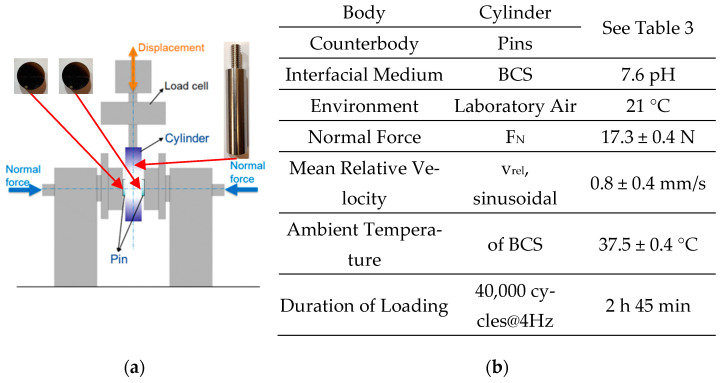
(**a**) Scheme of the fretting test rig. (**b**) Elements of the tribological system and loading parameters.

The fretting test rig was custom built, as published earlier [18,33,34,38]. The pins were clamped horizontally against the cylinder from opposite sides (Figure 2). All samples were electrically isolated from the rest of the test rig.

The contact area was fully immersed in 35 mL of bovine calf serum solution (BCS, 1000 mL of newborn calf serum and deionized water, 3.7 g of NaCl, 82.4 mg of EDTA, and 11.12 g of Trisaminomethan). The protein content was adjusted to 30 g/L by the ratio of newborn calf serum and deionized water, while the pH was tuned to 7.6 by adding HCl. In order to be able to compare this with our earlier experiments, the normal force F_N_ was again set to 17.3 ± 0.4 N, while the micromotion was achieved by sinusoidally oscillating the titanium cylinder with an amplitude of 50 ± 1 µm at 4 Hz. The temperature of 37.5 ± 0.4 °C was controlled by a heating circulator (Haake DC30, FisherScientific., Waltham, MA, USA), measured inside the test chamber by a thermometer (Fisherbrand Refrigerator/Freezer Plus Thermometer, FisherScientific., Waltham, MA, USA), and manually recorded. The tests were performed over 40,000 cycles (~8 m wear path) and repeated four to five times with a new triplet of two pins and one cylinder. The mechanical data, like the normal force F_N_, tangential (frictional) force F_T_, and displacement of the cylinder δ, were monitored and recorded for all cycles at 512 Hz (MTS-FlexTest60 Station Manager V5.986027 with the Multipurpose Elite V4.1.0.481, MTS Systems Corp., Eden Prairie, MN, USA). The frictional work per cycle W_d/c_, calculated from the area under the F_T_-δ hysteresis, was summed for all cycles to gain the accumulated frictional work W_acc_. In order to classify the fretting modes, the fretting regime criteria A, D, and B were derived from the measurements according to Fouvry et al. [56] and averaged over all cycles. For the ramping in and out of the hydraulic system, the first and the last nine cycles were not regarded for such analyses. The open corrosion potential (OCP) was measured with pins and a cylinder in contact under normal load for 60 min before and 30 min after the fretting tests. The samples in contact were used as the working electrode, a 0.5 mm Pt wire was used as the counter-electrode (Alfa Aesar, Ward Hill, MA, USA), and a Ag/AgCl electrode was used as the reference electrode (Gamry Instruments, Warminster, PA, USA). All OCP values were recorded at 1 Hz by means of a potentiostat (Gamry PCI4G750 Interface with Software Gamry Instruments Framework, V6.25).

The material combinations of the body (fluted cylinders) and counterbody (polished pins) used in this paper are given in Table 3, as well as the references for those combinations from earlier papers that were used for comparison.

#### 2.2.2. Microscopy and Raman Scattering Analyses

Depending on the required post-analysis, specimens were either sonicated (Branson 5800, Branson Ultrasonics, Brookfiled, CT, USA) for 10 min in 70/30 ethanol (for analyses of the surfaces with organic residues including the tribomaterial) or for 10 min in 2% enzymatic soap (Tergazyme, SigmaAldrich, St. Louis, MO, USA), followed by 10 min in 70/30 ethanol and 10 min in acetone (for the analyses of the surfaces without organic residues).

The tribological appearances of the contact areas and their surroundings, as well as local chemical analyses, were carried out by scanning electron microscopy (SEM) and energy-dispersive X-ray spectroscopy [57] (JSM-IT500HR with IT500HR V1.030, Jeol Technics Ltd., Peabody, MA, USA; Ultimax 65 with AZTEC 4.1 SP1, Oxford Instruments Nanotechnology Tools Ltd., Ann Arbor, MI, USA). The ceramic samples were sputtered with an AuPd coating of a 10 nm thickness before the SEM analyses (Cressington Sputter Coater 108auto/SE with the thickness controller MTM20, Cressington Scientific Instruments, Watford, UK).

Raman scattering analyses were performed with two different systems using laser excitations with the same wavelength of 532 nm (MonoVistaCRS, Spectroscopy&Imaging GmbH, Warstein, Germany; LabRAM HR Evolution, Horiba Scientific, Chicago, IL, USA). The laser power was adjusted to a few mW, avoiding laser-induced damages at the surfaces. The linearly polarized laser light was focused by confocal microscopes (BX51W1, L-BXFM-HR, Olympus, Shinjuku, Japan) on the samples, either with a 20 or 50 times magnifying objective. The scattered light was detected with spectrometers equipped with Si-based charged-coupled device cameras. The exposure time of the camera ranged from minutes to hours depending on the laser power, set between 3 mW and 80 µW. The scattering spectra were then compared to those from published references. Further details on the Raman scattering can be found in [58].

#### 2.2.3. Chemical Analysis of BCS and Enzymatic Soap

The 35 mL BCS beakers were stored at −21 °C, while the 100 mL soap beakers were kept at room temperature. After thawing the BCS samples, they were manually shaken for about 1 min. Finally, 1 mL was pipetted into smaller beakers for shipping at room temperature. Any occurring nanoparticles were not isolated from the ICP-MS liquids and became part of the measured metal content. The metal ion concentration within the serum as well as within the enzymatic soap solution was analyzed after the fretting test by means of inductively coupled plasma–mass spectroscopy (ICP-MS, Thermofisher iCAP TQ, Thermofisher, Lyon, France). An amount of 1 mL of the liquids were diluted by a factor of 20 in a mixture of 0.025 nHNO_3_, 0.05% Triton, and 1% alcohol. The measurements of Cr, Mo, and Ti were carried out in O_2_ mode, while, for the Co, the KED (kinetic energy discrimination) mode was chosen. All beakers were handed to the laboratory as blinded samples. All measured numbers represent the total metal contents of the liquids, while the fraction of the dissolved nanoparticles over metal ions is not known.

#### 2.2.4. Determining the Rates for the Generation of the Mechanically Mixed Layer and the Rates for the Wear Loss

In order to distinguish between the generated wear products remaining in the tribosystem and those being ejected (representing the wear loss), the metal concentration of both the BCS and enzymatic soap was measured. Here, the BCS samples represent the ejected wear loss, while the soap samples provide the contributions to the generated mechanically mixed layer. Still, these analyses cannot render the full quantitative information. Some general limitations appear by the following aspects:

- BCS already contains high amounts of Fe and Al. Thus, those elements cannot be used for the analyses of the contributions of AHNS or ZTA to the tribomaterial or wear loss.
- Not all contaminants within the hospital’s tab water were removed by reverse osmosis, the process used for the DI water. Thus, the measured gross Ti amounts were corrected by the measured amount in the BCS blanks of 30.87 ppb, which was carried over from the DI water and the constituents within the respective NBCS lot.
- In ICP-MS, the plasma dissolves all matter, resulting in a loss of any quantitative value for the particles that were ejected from the tribosystem. Hence, the fraction of particles to ions cannot be determined.

The numbers of elements within the serum and soap, therefore, only allow for a qualitative ranking of their contributions to the mechanically mixed layer and wear loss under ultra-mild fretting wear. The obtained rates were normalized to the measured length of the wear path. The procedure was as follows:

- Deduct 30.87 ppb from all gross Ti values to account for the contamination found in the DI water.
- Relate the net concentration value to the weight of the 35 mL serum and 100 mL soap sample containments, respectively, in order to obtain the absolute values in ng.
- Relate these absolute values to the actually measured lengths of the wear paths and gain a rate in ng/m. This was primarily performed to account for some experiments that ran for 40,000 cycles and others that ran for 50,000 cycles.

Since Co, Cr, Mn, and Ti are released dependent on the alloys’ compositions, a direct comparison would not describe the relative behavior. Thus, the obtained ng/m values were further normalized for Co, Cr of the Co alloy, Cr and Mn of the AHNSs, and Ti by the factors 0.65, 0.3, 0.18, 0.125, and 0.9, respectively, to account for their abundance within each alloy. By adding all these contributions, one gains the normalized gross generation rates of the mechanically mixed layers g_MML_ and of the material loss w_FC_ (by the fretting corrosion) of the whole tribosystem. Nevertheless, one cannot gain information about absolute amounts nor about the volume or weight fraction of the organic constituents of the generated mechanically mixed layer or the ejected mass.

## 3. Results

### 3.1. Fretting Regime

According to the Hertzian model of two curved surfaces, the semi-length of the elliptic nominal contact area parallel to v_rel_ is about 1.96 mm [18,59] and, therefore, larger than the amplitude of 50 µm. Thus, all experiments fulfil the criterion for a fretting contact in general [56]. The fretting regime is characterized by the work (A) and sliding (D) ratios, as well as the system-free parameter (B) [56]. These are computed by the F_T_-δ hystereses and compared to the transition values A_t_, D_t_, and B_t_. A, D, and B are always larger than A_t_, D_t_, and B_t_ (Table 4). Thus, all tests fulfil the criterion for gross slip fretting.

### 3.2. Frictional Work

**Figure 3:**
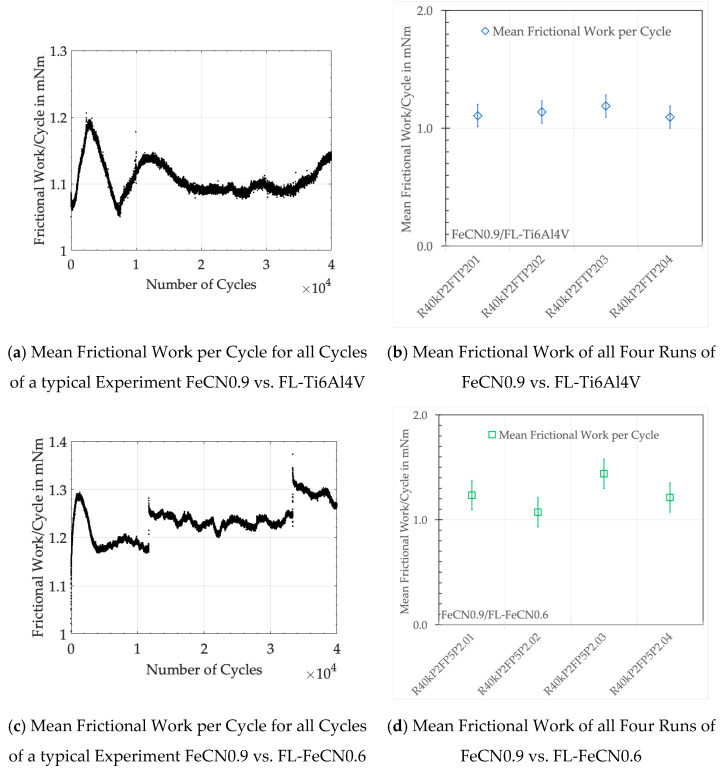
Mean frictional work of experiments with FeCN0.9 runs against (**a**,**b**) fluted Ti6Al4V and (**c**,**d**) fluted FeCN0.6.

Figure 3 shows examples of the frictional work per cycles of all cycles of a single run of steel vs. Ti alloy and steel vs. steel (Figure 3a,b), as well as the mean values of four runs of these couples (Figure 3c,d). Obviously, the frictional work is not a constant and changes between cycles, which is related to the continual changes in the nature of the contacts and the size of the real contact area as well as the behavior of the wear products within the interface. Despite this scattering, W_d/c_ remains close to its mean value if averaged over all cycles. Table 5 wraps up those numbers for all experiments as well as those of the accumulated dissipated frictional work over all cycles W_acc_.

These numbers represent the balance between all incidents that might raise or lower the friction. It appears that the AHNS vs. FL-Ti alloy tends to smaller values, while the AHNS vs. FL-Co alloy tends to larger ones, which is comparable to the Co alloy vs. FL-AHNS. The frictional work of the self-mating AHNS/AHNS ranges about in between, while the ZTA/FL-AHNS couples show the lowest frictional values. This is quite surprising, as the formerly published values of W_acc_ with ZTA/FL-CoC0.06 and ZTA/FL-Ti6Al4V couples were larger at 54.16 ± 2.58 Nm and 71.85 ± 2.88 Nm, respectively [34].

### 3.3. OCP Drop (ΔOCP) during Fretting Experiments

**Figure 4:**
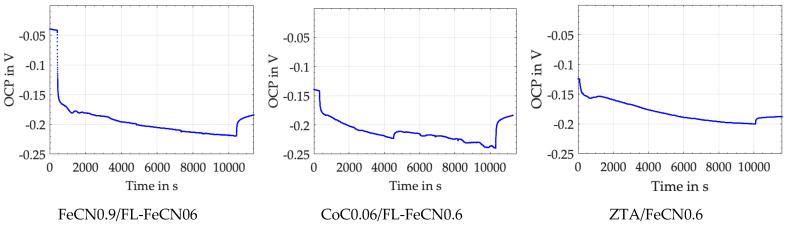
OCP over the entire duration of the fretting experiments for different pin materials against FL-FeCN0.6.

Figure 4 depicts the OCP drop (ΔOCP) at each start of the fretting experiment, as well as its steady-state behavior, which begins after about 500 to 1000 cycles. The immediate drop is based on the mechanical disruption of the passive layers, while during the steady state, the depassivating (tribocorrosion) and repassivating (tribo-oxidation and mechanical mixing) incidents compete into a more or less stable equilibrium. The final increase represents the repassivation of the surfaces after the experiments have been stopped. Like it was for friction (integrating over all mechanical incidents), one cannot distinguish between single chemical incidents by the OCP. Still, the slow decrease during the steady state—here, about 0.05 V in 39,500 cycles—is quite likely related to an increase in the real contact area. If a larger area is depassivated during cycling, the OCP drops for an increase in the number of ions released.

Table 6 wraps up the ΔOCP, which is mostly around 0.3 V for AHNS/FL-Ti6Al4V and AHNS/FL-CoC0.06, but smaller for all couples against FL-AHNS.

If we compare these values with those of ZTA/FL-CoC0.06 at 0.32 ± 0.01 V and ZTA/FL-Ti6Al4V at 0.52 ± 0.04 V, published earlier [34], we can assume that this electrochemical feature can only be governed by the metallic side of the couples. It appears that the ranking cannot be connected to the repassivation results, e.g., from the electro-impedance spectroscopy (EIS) experiments [33,60,61]. We can only argue at this point that the repassivation behavior on a polished surface in EIS experiments is completely different from that of a severe plastically deformed and mechanically mixed layer inside the crevice of a fretting corrosion contact interface. From the ΔOCP, we can only read that the smaller, the less ions are released and, therefore, the better. In a rough approximation as to this criterion, we could write that AHNSs would outperform the Co alloy [54] and that Ti6Al4V would be the worst if run against an alumina-based ceramic [34]. But, if FL-Ti6Al4V is run against the AHNS (Table 6), the ΔOCP values do not follow that trend. Thus, we cannot explain the elementary processes that drive such behavior at this point in our analyses. Furthermore, we do not know the real active areas for depassivation and repassivation, as well as the different kinetics of tribocorrosion and tribo-oxidation on each side of the contact.

### 3.4. Wear Appearances of Polished FeCN0.6 against Fluted Ti6Al4V Cylinders (AHNS/Ti Alloy)

**Figure 5:**
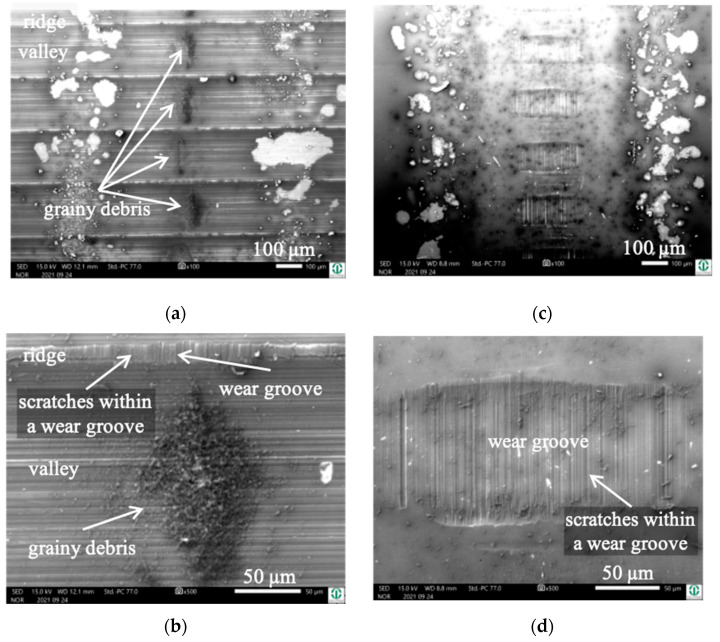
Wear appearances on a fluted Ti6Al4V cylinder (**a**,**b**) after 40,000 cycles against FeCN0.6 (**c**,**d**). The samples have been sonicated in ethanol.

Due to the fact that the wear appearances of the fluted Ti6Al4V against FeCN0.9 and FeCN0.6 looked the same, we only present the analyses of the AHNS/FL-Ti6Al4V couples together with some examples of both as to specific aspects. Figure 5 depicts the surfaces of the fluted Ti6Al4V cylinder (Figure 5a,b) and a polished FeCN0.6 pin (Figure 5c,d) after the fretting test. The samples were sonicated in ethanol in order to remove some loose debris but to keep most of the grayish and partly grainy tribomaterial (third bodies). Still, some of it spalls off during sonication, as can be seen by the lighter whitish areas on both bodies. It should be mentioned here that the horizontal scars within the valleys are machining marks, while any vertical scars or scratches are parallel to v_rel_ and are generated by friction and wear.

Due to the two convex surfaces, the Hertzian [59] gross contact area is elliptic, with a semi-contact length of 1.96 mm for, e.g., FeCN0.6/FL-Ti6Al4V. The width is zero at both ends and has a theoretical maximum value in the center of 52 µm. With the fluted topography (R_sm_ = 190 µm), about 20 ridges could theoretically be in contact over the entire length of the gross contact area. Now, the tribological loading and the generation of wear grooves bring about a marked change, with about 51 ridges in contact over approximately a 10 mm contact length. The tip radius within the 2 to 5 µm range and the shape of the circumferential ridges only allows for a quite narrow contact parallel to v_rel_ and a much wider one perpendicular to it. CWLM analyses brought about dimensions ranging from 9 to 17 µm parallel to v_rel_ and 116 to 286 µm perpendicular to it for, e.g., FeCN0.6/FL-Ti6Al4V. The CoC0.03/FL-Ti6Al4V couples, published earlier [38], bring about similar values, with 5 to 11 µm and 129 to 229 µm, respectively.

Due to the unknown contact stresses, strains, and fluid characteristics of the tribomaterial inside such a particle-generating and debris-laden rough interface, any contact mechanical modeling or simulations will not be a part of this paper. We also did not quantify the depth of the wear grooves, for under ultra-mild wear, these values are within the range of the circumferential roughness on top of the machined ridges as well as of the overall waviness of the polished pins. Additionally, there were pile-ups of tribomaterial or plastically deformed metal at the rims of the grooves, which did not allow for the finding of an absolute surface reference level to be defined as the zero level. Thus, with our specimens, the depth of the grooves cannot be separated from other surface features near or outside of the contact areas. As a result, we are not able to give the quantities of the grooved volumes of both bodies.

Cylinder side: On the fluted cylinder side, piles of grainy (Figure 5a,b) wear debris can be seen inside the valleys partly covering the machining marks. The wear grooves on top of the ridges (Figure 5b) show vertical scratches, while it cannot be distinguished whether they stem from microcutting or microploughing. This is also true for the areal wear grooves on the surface of the polished pins (Figure 5c,d), which show such scratches as well.

**Figure 6:**
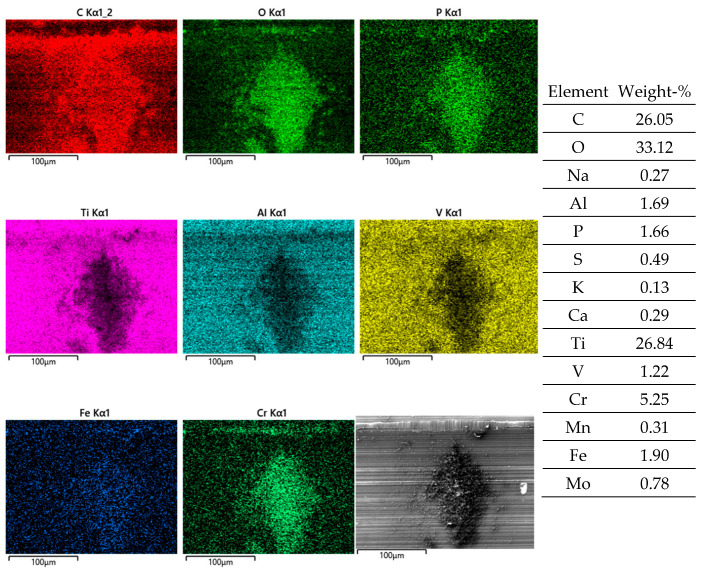
EDS scans of a contact ridge and grainy debris on fluted Ti6Al4V cylinder. The samples have been sonicated in ethanol.

Obviously, the grainy debris sticks rigidly to the surface of the cylinders, as it is still visible within the valleys even after sonicating in ethanol. This allowed for EDS scans and point analyses (Figure 6), and revealed elements of the Ti alloy (Ti, Al, V) as well as O, Cr, and P from the counterbody and the lubricant. On top of the contact ridges, there are substantial remains of C and O from the lubricant and its reaction products, like oxides. Cr oxides can be assumed from the Cr/Fe ratio of 2.8 being much larger than that of the alloy of 0.26. Additionally, Ti oxides are possible, which will be later elucidated by the Raman scattering results. The elements like C, O, P, Na, K, and Ca can be found on top of the ridges within the grooves as well as within the valleys. Remarkedly, there is nearly no grainy debris left inside the grooves on top of the ridges of the cylinders as well as inside the areal grooves on the pin side (Figure 5).

**Figure 7:**
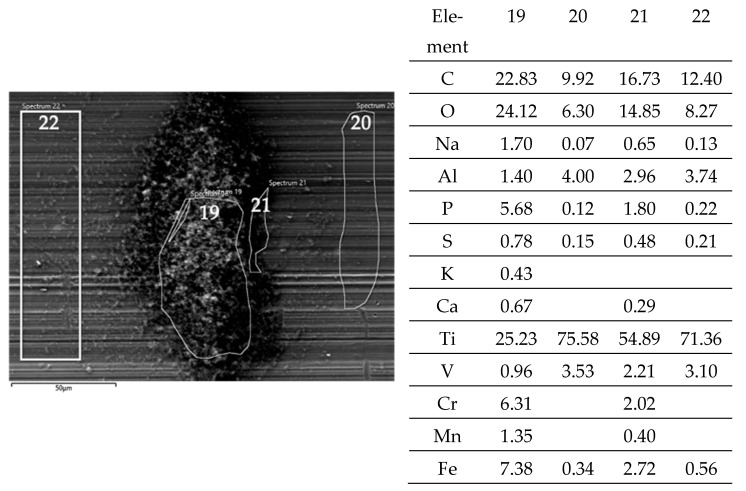
Pile of grainy debris (areas and spectra 19 and 21) within a valley (areas and spectra 20 and 22) of a fluted Ti6Al4V cylinder after the fretting test against polished FeCN0.9. The samples have been sonicated in ethanol. Blank cells within this and all further EDS tables mean “not detected”.

A close look at one of the debris piles by the EDS allows for a comparison to the adjacent steel surface after the fretting test and sonication in ethanol. The layer on top of the valley’s surface (Figure 7; Spectra 20, 22) shows the distinct remains of the lubricant by C, O, P, and S, while the high amounts of Ti stem from the background signal. The Al/Ti ratio is about 0.05 and similar to that of the alloy. There is no Cr and Mn, while the Fe signals of 0.3 and 0.6% originate from the Fe content of the BCS. At the rim of the pile of debris (Figure 7, Spectrum 21), C, O, P, and S, as well as Ca from the lubricant increase as well, with the background signal of the Ti alloy (Al/Ti ratio = 0.05) being still quite strong. Cr and Fe increase as well, while the Cr/Fe ratio of about 0.7 points towards oxides. The pile of debris (Figure 7, Spectrum 19) reveals the highest values of C, O, P, S, and Ca from the lubricant, as well as of Cr and Fe. The Cr/Fe ratio of 0.85 being larger than that of the alloy points towards oxidized debris from the pin side remaining on the cylinder. Whether the Ti oxide particles are part of the debris might appear quite likely but cannot be read from the EDS scans, as the Al/Ti ratio is still similar to that of the alloy.

**Figure 8:**
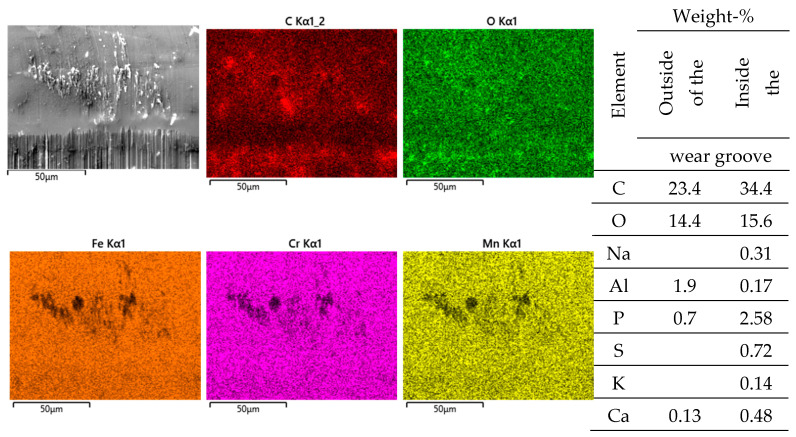

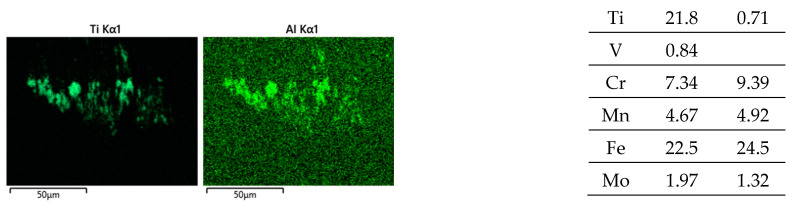
EDS scans of a contact ridge and grainy debris on the FeCN0.6 pin. The samples have been sonicated in ethanol.

Pin side: EDS scans reveal, outside of the wear grooves (Figure 8), elements of the lubricant, while, in between the wear grooves, Ti- and Al-rich particles (an Al/Ti ratio similar to that of the alloy) can be found. Thus, these particles have quite likely been generated by microcutting and represent chips from the cylinder side.

Inside the wear grooves, one finds Fe, Cr, Mn, and Mo (Figure 8), as well as localized remains of tribomaterials, including C, O, Na, P, S, K, and Ca. The Cr/Fe ratio of about 0.38 is only a little bit larger than that of the alloy and by far not as high as on the cylinder side. Thus, oxide particles cannot be assumed, as the background noise from the alloy appears to overlap with these measurements.

### 3.5. Wear Appearances of Polished FeCN0.6 against Fluted CoC0.06 (AHNS/Co Alloy)

**Figure 9:**
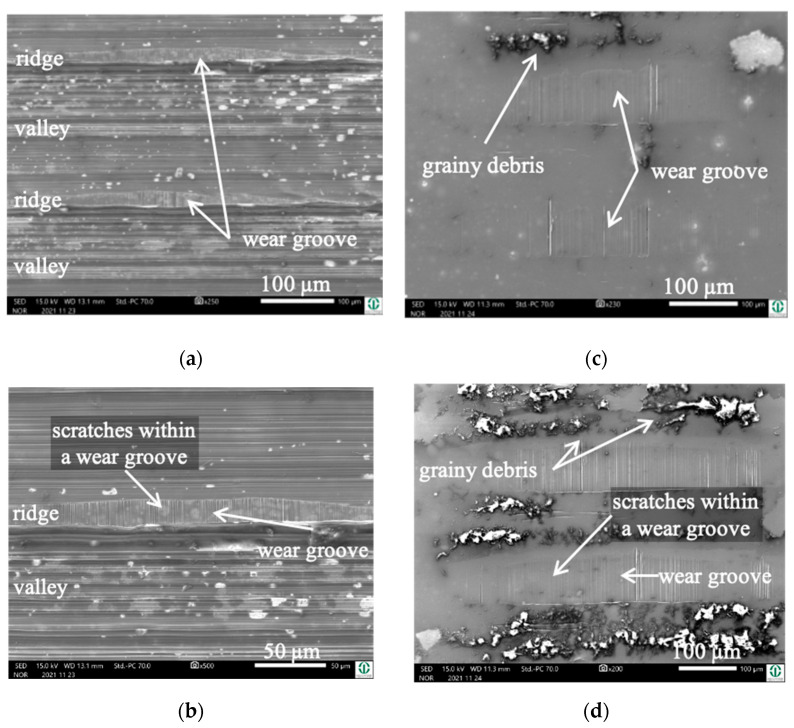
Wear appearances on a fluted CoC0.06 cylinder (**a**,**b**) after 40,000 cycles against polished FeCN0.6 pins (**c**,**d**). The samples have been sonicated in ethanol.

**Figure 10:**
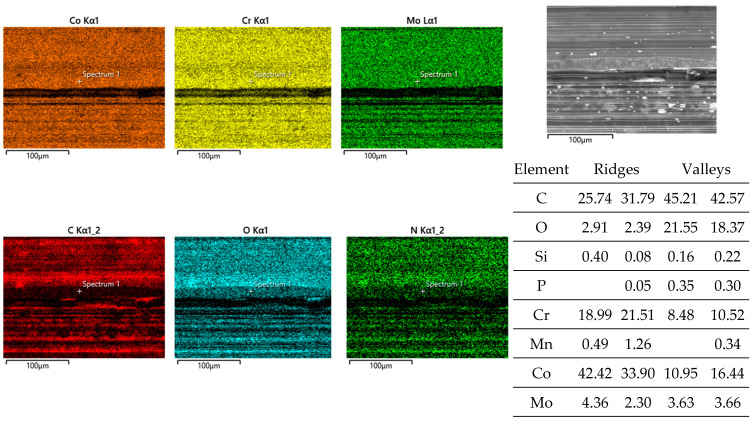
EDS scans of a contact ridge and grainy debris on a fluted CoC0.06 cylinder. The samples have been sonicated in ethanol.

By comparing Figure 5 and Figure 9, it becomes obvious that—after sonication in ethanol—most of the grainy debris remains on the pin side (Figure 9c,d), while there are no marked piles of debris inside the valleys of the fluted topography (Figure 9a,b and Figure 10). Apart from that, the wear appearances are quite similar on both bodies, with narrow wear grooves on top of the ridges and areal ones on the polished pin. Both show scratches, suggesting microcutting and/or microploughing. 

**Figure 11:**
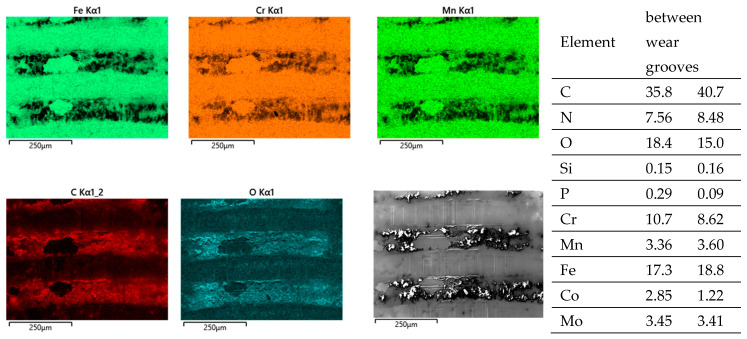
EDS scans of grainy debris between the wear grooves on the FeCN0.6 pin. The samples have been sonicated in ethanol.

Pin side: EDS scans reveal background noise of the steel with Fe, Cr, Mn, and Mo, as well as the localized remains of the lubricant by C and O (not shown here). In between the areal wear grooves, there are further indications of the lubricant by C, O, and P (Figure 11). From the Cr/Co or Cr/Fe ratios, we cannot read anything, as the EDS cannot distinguish the specific source of Cr. Still, for both alloys, the ratios would be smaller, which might render a hint on the Cr-based oxidic wear debris.

### 3.6. Wear Appearances of Polished CoC0.06 against Fluted FeCN0.6 Cylinders (Co Alloy/AHNS)

**Figure 12:**
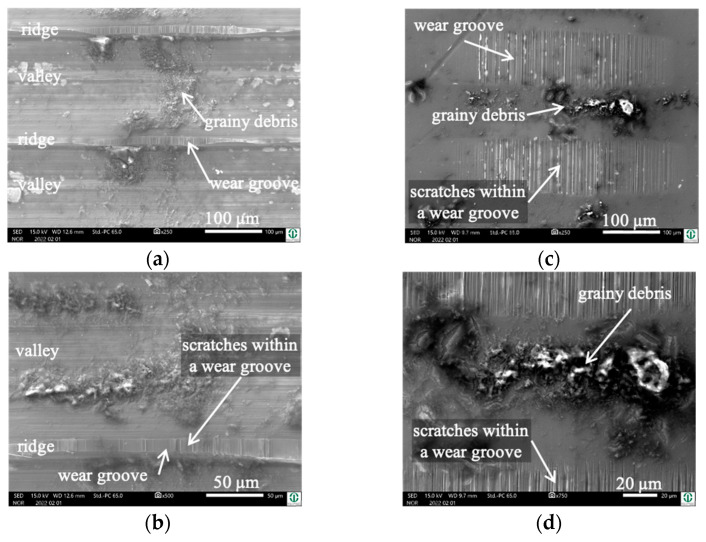
Wear appearances on a fluted FeCN0.6 cylinder (**a**,**b**) after 40,000 cycles against CoC0.06 pins (**c**,**d**). The samples have been sonicated in ethanol.

**Figure 13:**
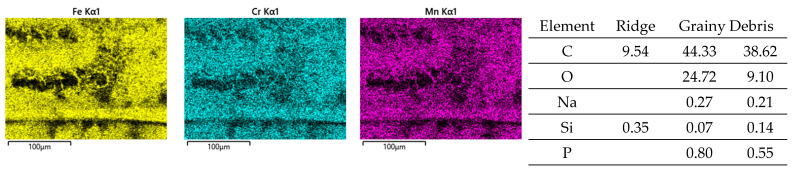

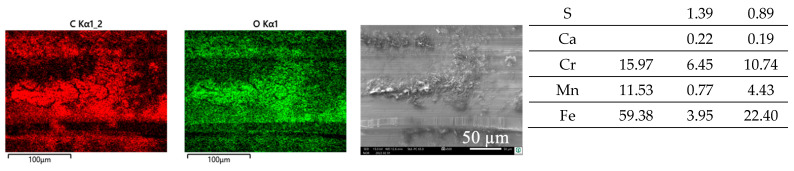
EDS scans of a contact ridge and grainy debris on a fluted FeCN0.6 cylinder. The samples have been sonicated in ethanol.

**Figure 14:**
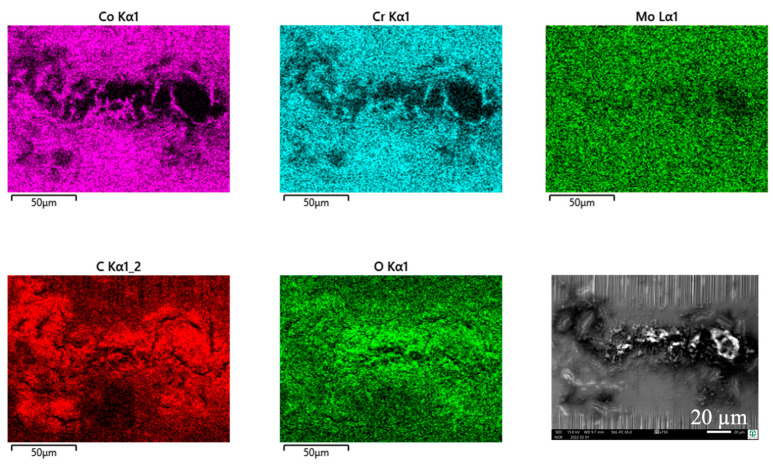
EDS scans of grainy debris on a polished CoC0.06 pin. The samples have been sonicated in ethanol.

By switching body and counterbody, there is no distinct change in the wear appearances. The wear grooves of both bodies reveal scratches parallel to v_rel_ (Figure 12a–d). The grainy tribomaterial piles up within the valleys (Figure 13 C, O) on the cylinder side and in between the contact areas on the pin side (Figure 14 C, O).

Cylinder side: Spot analyses of the grainy debris reveal elements of the lubricant (C, O, Na, P, S, and Ca) and those of the alloy (Fe, Cr, and Mn) (Figure 13). Again, the Cr/Fe ratios from 0.4 to 1.6 are larger than that of the alloy of 0.29 and indicate oxidic debris. On top of the ridges, there is still some C, pointing towards a thin layer of the remains of the lubricant, as the Cr/Fe ratio from the underlying material resembles that of the alloy.

Pin side: On the pin side, the appearances are quite similar in general. Within the wear grooves, there is still a thin layer of C as the remains of the lubricant, while the Cr/Co ratio is that of the alloy. The grainy debris in between the wear grooves shows C, O, P, S, and Ca from the lubricant and Co and Cr from the pin’s alloy (Figure 14, Table 7). Traces of Fe, Mn, and maybe also Cr from the cylinder indicate a very small portion of reaction products from the cylinder side. Thus, most of the Cr should come from the Co alloy and indicate Cr oxides for the Cr/Co ratio ranging between 0.59 and 1.6 (larger than that of the alloy) depending on the local thickness of the debris layer, and, therefore, the included fraction of the background signal.

### 3.7. Wear Appearances of Polished FeCN0.9 against Fluted FeCN0.6 Cylinders (AHNS/AHNS)

**Figure 15:**
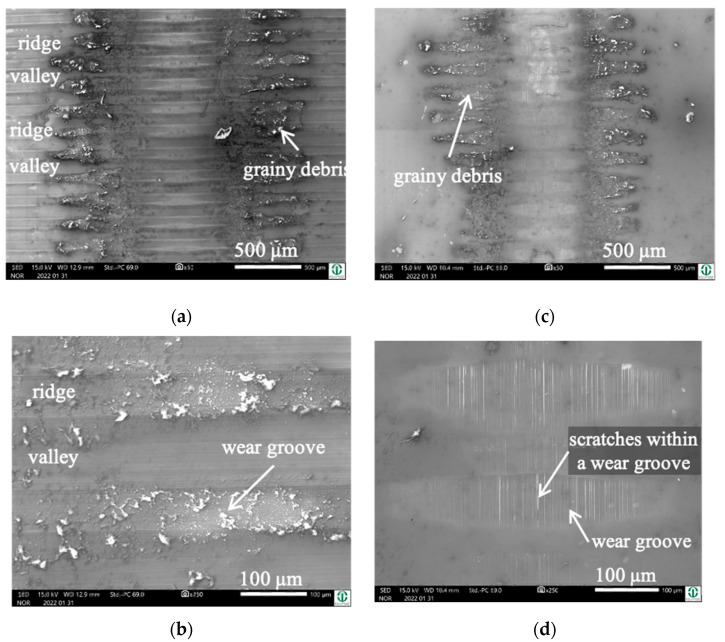
Wear appearances on a fluted FeCN0.6 cylinder (**a**,**b**) after 40,000 cycles against polished FeCN0.9 pins (**c**,**d**). The samples have been sonicated in ethanol.

The wear appearances of this self-mated couple look slightly different, in that the debris is found on top of the ridges and pushed out to both sides of the wear track on the cylinder (Figure 15a,b) as well as on the pin side (Figure 15c,d). The wear grooves show vertical scratches as a wear appearance, as it was for the former couples.

**Figure 16:**
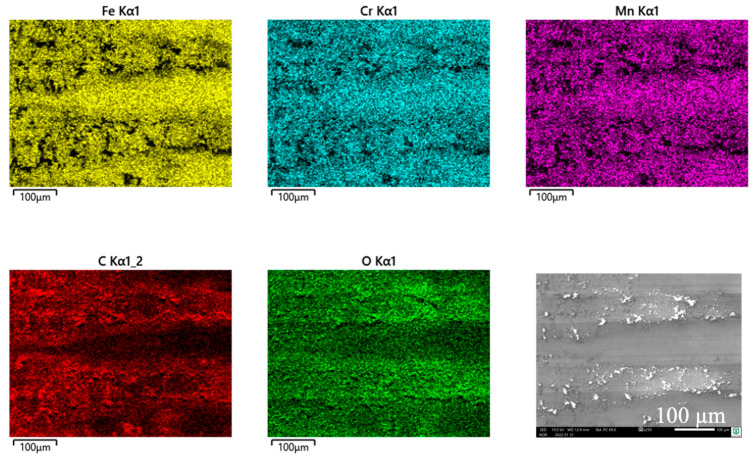
EDS scans of the debris pushed out to both sides of the contact area of the fluted FeCN0.6 cylinder. The samples have been sonicated in ethanol.

**Figure 17:**
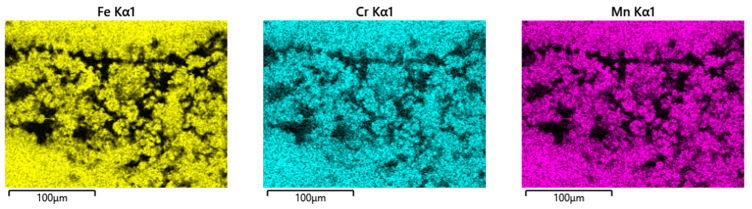

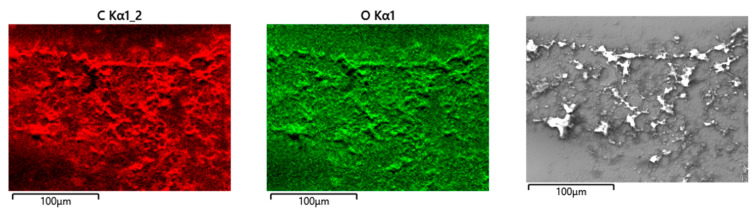
EDS scans of grainy debris on a polished FeCN0.9 pin. The samples have been sonicated in ethanol.

Cylinder side and pin side: In this self-mated couple, the debris appears quite similar on both bodies (Figure 16 and Figure 17), which is supported by the EDS analyses shown in Table 8 and Table 9.

The white debris reveals elements of the lubricant, like C, O, P, S, and Ca, as well as a Cr/Fe ratio that is much larger than that of the alloy. Thus, again, Cr-based oxidic wear debris can be assumed here.

### 3.8. Wear Appearances of Polished ZTA against Fluted FeCN0.6 Cylinders (ZTA/AHNS)

**Figure 18:**
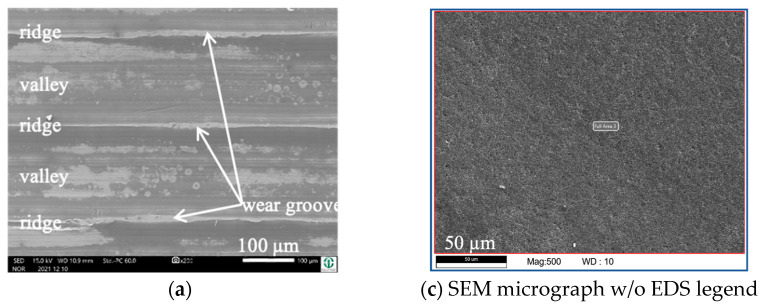

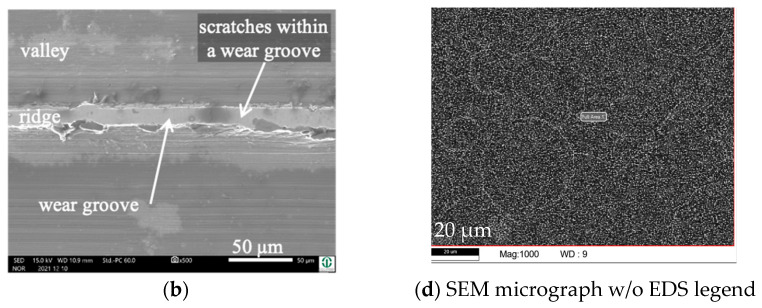
Wear appearances on a fluted FeCN0.6 cylinder (**a**,**b**) after 40,000 cycles against ZTA (**c**,**d**). The samples have been sonicated in ethanol.

The wear grooves on top of the ridges show lesser and finer scratches compared to the metal/metal couples (Figure 18a,b). The debris stays attached to the rims of the ridges, while the valleys show the remains of the tribomaterial (darker gray areas), which spalls off during sonication in parts (lighter gray areas). As shown earlier [34] for the ZTA/FL-Co alloy couples, there are no obvious wear appearances on the ceramic side of the contact. Within the contact area (Figure 18c,d), the surfaces look like the polished materialographic (Figure 1c) samples, showing the typical microstructure of such ZTA ceramics.

**Figure 19:**
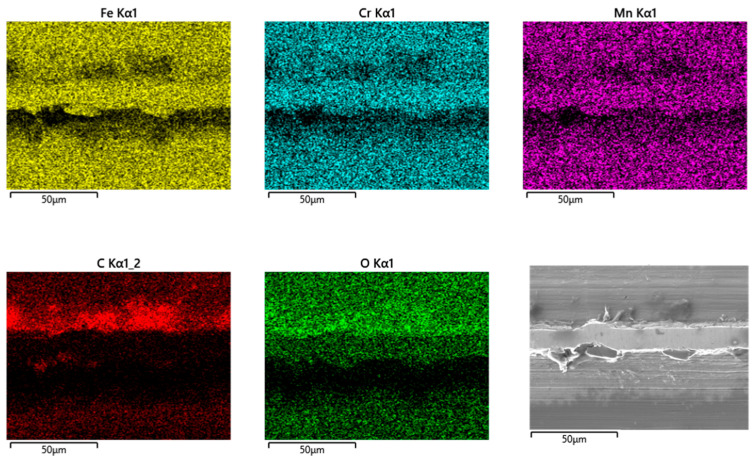
EDS scans of a contact ridge and grainy debris on fluted FeCN0.6 cylinder. The samples have been sonicated in ethanol.

EDS scans and spot analyses revealed C and O as the constituents of the lubricant on top of the ridge (Figure 19, Table 10), and additional P and Ca within the debris (Table 10). The Cr/Fe ratios are close to that of the alloy and can be related to the background noise.

The EDS analyses of the pins just rendered the chemical composition of the ZTA ceramic, a slightly higher C content from remains of the lubricant. Apart from that, there were absolutely no indications of any measurable transfer of materials from the cylinder nor the remains of any grainy debris.

### 3.9. Raman Scattering Analyses of the Tribomaterial of AHNS/FL-Ti6Al4V Contacts

The metallic–ceramic–organic composite, also known as tribomaterial, within the contact interface stems from body and counterbody, as well as their reaction products with the lubricant. For its nanostructure and the chemical elements involved, the EDS can only render a first glimpse of the specific phases. Thus, Raman scattering analyses were used to specify these constituents as well as the alterations within the molecular structure of the BCS. The latter, as well as those of the Co and Ti alloys, have been analyzed by TEM, APT, and RS, and the results are either published or submitted for review [31,38,62,63] and will not be elucidated further in this paper. Thus, we will focus on the reaction products of the AHNS FeCN0.9. Because of the identical EDS findings, these analyses were limited to the pins’ surfaces, which allowed for easier handling than with the cylinders. The RS analyses indicated the spectra of adsorbed O_2_ and N_2_ molecules in all positions, which stem from the laboratory atmosphere and which will be neglected in the following.

Before a fretting test, and after sonication in ethanol and drying in laboratory air, AHNSs showed MoO_3_ and Cr_3_O_8_ spectra from the passive film. If immersed in BCS for 3 h at 37 °C, one finds additional proteins like native albumin and globulin, as well as phospholipids, besides other typical amino and fatty acids on the surface.

After the fretting experiment, the picture changes with respect to the position of the analyses outside or inside the contact area (Table 11).

Within the immediate contact area, which includes the ridges and valleys on the cylinder, as well as the wear grooves and areas between them on the pin, there are additional reaction products from the base and alloying elements of both bodies, e.g., Fe_2_O_3_ or MnMoO_4_ from the AHNS or TiO_2_ from Ti6Al4V. Besides the typical constituents of the BCS, there are additional denatured and cleaved proteins, as well as sp^2^-hybridized C, as already found in earlier in vivo, in vitro, and retrieval studies after tribological loading [62].

The pushed-out material outside of the immediate contact area was generated by the tribosystem containing all metallic, ceramic, and organic constituents of the interfacial medium. Nonetheless, it has not been ejected yet. Still, we can assume that the ejected fraction stems from this pushed-out material and, therefore, contains all the metallic, ceramic, and organic matter of the final wear loss.3.10. Metal Ion Concentration within the Soap and Serum

#### 3.9.1. Metal Ion Concentration within the Soap

The largest contribution to the tribomaterial by far stems from the Ti cylinders (Table 12), as has been reported before for other material couples [20,39,44]. Far less comes from Co or AHNS cylinders, represented by Co and Cr or Mn and Cr, respectively. Interestingly, the Mn content is within the same range between 9 and 18 ppb, independent of the specific couple, while the Co and Cr show two to four times larger values, ranging from 34 to 45 ppb, with two exceptions. One is for the FeCN0.9/FL-Ti6Al4V couple and one is for the self-mating FeCN0.9/FL-FeCN0.6 couple, with 12 and 16 ppb, respectively.

Before we use this for any ranking, one has to consider that some of these elements, like Ti and Cr, are more prone to generate reaction products than Co or Mn. In addition, Ti alloys contain relatively much more Ti than there is Co in Co alloys or Cr and Mn in steels, which requires normalization, as described in the Methods section. Finally, for the AHNS/Co alloy couples, we cannot distinguish the amount of Cr coming from the Co and from the steel side. With these couples, the exchange of cylinder and pin materials makes no difference in the contributions of Co and Cr, but it does in the case of Mn.

#### 3.9.2. Metal Ion Concentration within the Serum

The numbers above change markedly for the material ejected from the tribosystem (Table 13). Now, Co is the most distinct contributor to the material loss. This is true for both the cylinder and pin material in contact with the AHNS. The Ti values are minimal, not exceeding 3 ppb, while the Cr and Mn range from 5 to 32 ppb and 14 to 37 ppb, respectively. The AHNS as cylinders show the largest Mn values in combination with the lowest for Cr, while the AHNS as pins contribute much less Mn. Interestingly, the AHNS/Ti6Al4V couples release less Cr into the serum compared to the AHNS/Co alloy ones. This might lead to the assumption that the higher Cr release of the latter is mainly connected to the wear loss of the Co alloy rather than to that of the steel.

Again, any ranking or comparison with the values of the soap can only be performed after some normalization, which will be carried out and elucidated within the Discussion section.

## 4. Discussion

### 4.1. Preliminary Remarks and Definition of Terms

We do not intend to show any absolute quantitative tribological data, but rather the tendencies of the tribological behavior under ultra-mild fretting wear conditions. Thus, formerly published data are included when needed to better illustrate such tendencies. We also found earlier that the depth or volume of the wear grooves do not relate at all to the amount of material lost from the tribosystem. It certainly could appear that such volume might be relatable to the amount of tribomaterial generated, which remains within the contact area. Still, parts of it spalled off during sonication in ethanol, which is another reason to refrain from quantification. Thus, we follow our former strategy to rank the tribological behavior and relate this to the mechanisms and submechanisms characteristic for ultra-mild fretting wear.

**Figure 20:**
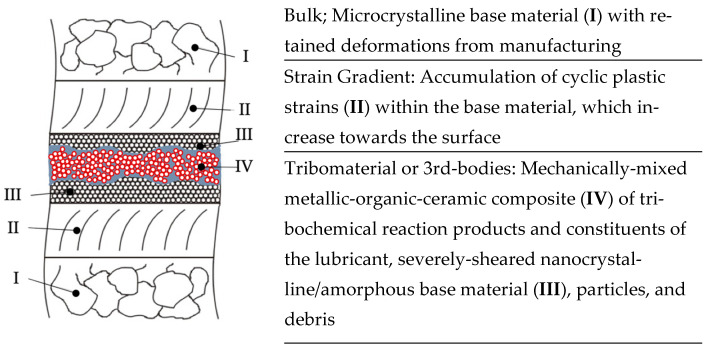
The graded structure of sliding interfaces according to reference [30].

Another limitation comes from the fact that the severely sheared tribomaterial (or third bodies) is a graded structure (Figure 20). Close to the contact surface, the fraction of reaction products and the remains of the lubricant are much larger than that close to the interface to the strain gradient. Thus, during sonication in soap, we mostly removed that part, which contains organic constituents. Still, the severely plastically deformed nanocrystalline base material that is not mixed with organic matter remains on the surface during soap cleaning. As a result, there are unknown parts of the tribomaterial that cannot be found in the soap samples. Thus, our normalized rates from the soap samples only apply to that fraction of the tribomaterial that contain organic constituents. We could have called this fraction “the loose third bodies”, allowing velocity accommodation, but this would violate fundamental aspects of the third-body approach (which includes the whole nanocrystalline layer) [20,64]. Thus, and in order to distinguish between the different graded structures of the tribomaterial, we will use “mechanically mixed layer” (MML) for this fraction of the tribomaterial that is captured by the soap treatment.

### 4.2. Wear Appearances and Acting Wear Mechanisms and Submechanisms

The wear appearances with the AHNS as the body or counterbody mainly resemble those we found earlier with different couples of similar materials [18,33,34,35,38]. The ridges on the cylinder side show scratches that point towards the submechanisms of abrasion, namely, microcutting and microploughing [30,65]. From this, we can conclude that there must have been particles generated within the interface. The EDS revealed the chemical alterations of these surfaces, which can be related to the submechanisms of tribochemical reactions, called tribo-oxidation and mechanical mixing. The latter submechanism is connected to the severe plastic deformation on different length scales, which, besides microcutting and microploughing, requires frictional work. Now, all of these submechanisms are known to be able to generate wear particles, as well as all kinds of layer-like and granular tribochemical reaction products [66,67]. These might remain within the interface and not necessarily leave the tribosystem immediately. In combination with the other constituents of the lubricant, they create a nanostructured interfacial medium from metallic, ceramic, and organic compounds that can act as boundary lubricants and protect the surfaces from direct metal–metal contacts [20,29,36,68,69].

### 4.3. Frictional Work

**Figure 21:**
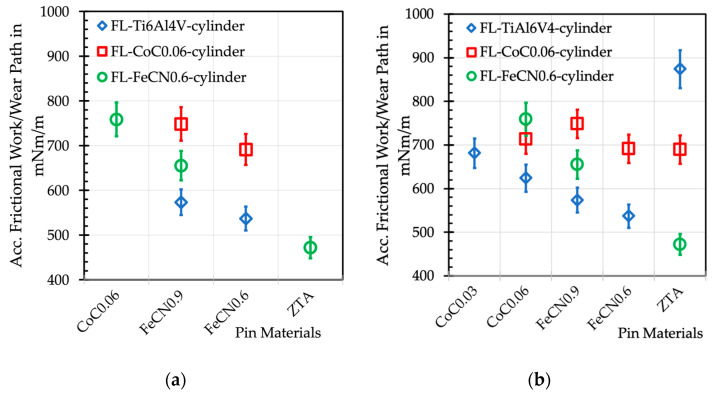
Accumulated frictional work per wear path W_acc/l_ in mNm/m of the different combinations of materials. (**a**) W_acc/l_ of the fluted couples, this work; (**b**) W_acc/l_ of all couples tested [20,39,44].

Measuring friction in a tribometer always integrates lots of different effects into a single number. Thus, it is impossible to read the influence of single incidents (adhesion, deformation, and interlocking) of friction mechanisms from such friction values. Nevertheless, if we find marked differences in the accumulated frictional work in fretting experiments, we should discuss the reasons for it and try to relate it to the appearances of the surfaces and the interfacial media after tribological loading. In a first and simple approach, we may focus on deformation as a substantial contribution to friction for all wear grooves, as well as the remains of tribomaterial connected to plastic deformation—or the materials’ resistance to it—within the real contact area. In order to be able to compare the results from the former fretting experiments with the newer ones, Figure 21a depicts the accumulated frictional work over all cycles related to the length of the wear path for the material combinations of this paper, while Figure 21b includes all the couples tested so far. It appears as if the values scatter around 700 mNm/m whenever Co alloys are involved as cylinder or pin materials, while the AHNS/FL-Ti6Al4V couples allow for values below 600 mNm/m.

Still, the couples with the ZTA pins differ most markedly depending on the fluted cylinders’ alloy. On the basis of the wear appearances, we can certainly consider that, with these couples, the plastic deformation is limited to the metal side. Thus, the higher strength of the CoC0.06 alloy would result in higher friction compared to the FeCN0.6. But the ZTA/FL-Ti6Al4V couple does not fit into such a relation. This is based on the reported [34] additional contribution of the distinct materials transfer from the Ti to the ZTA side by microweldings, a submechanism of adhesion, that we could not find for the couples with the Co- or Fe-based alloys.

At a first glance, for the other couples, we can just consider microcutting or microploughing as mechanisms, and that the ranking is related to the strength or cold-working capability of the metals [70]. But we do not know the contribution from the body or counterbody for the different materials in contact, as well the differences in the size and shape of the wear grooves. Additionally, the mechanical properties are measured under an average equivalent strain rate of about 10^−2^ s^−1^, while, within the severely plastically deformed tribomaterial, the average strain rates are more than 10 orders of magnitude larger [71]. From the severe plastic deformation (SPD) literature that we know of, such strain rates require distinctly different deformation mechanisms than under hardness or tensile tests [72]. Finally, the differences in the stacking fault energies [73] or the tendency of the materials to form shear bands might have an influence [74], as well as other micro- and nanostructural effects that are still under investigation [25,75,76]. Including the contribution of the tribomaterial appears impossible, for we do not know its structural, mechanical, and fluidic properties under high shear rates at all. Thus, any relation of ranking friction to certain material properties is purely arbitrary, if not useless.

Now, many papers relate the frictional work under fretting to the wear loss assuming that the mechanical-dominated wear mechanisms are characteristic for this. We have shown in the past that this does not apply to ultra-mild fretting wear, under which the chemical-dominated wear mechanisms govern the wear loss. Still, it would be interesting to know whether the frictional work at least influences the generation of tribomaterial.

### 4.4. The Normalized Gross Generation Rate of the Mechanically Mixed Layer g_MML_

Since we cannot give any quantitative amount of the tribomaterial generated, we used its main chemical elements from the ICP-MS analyses of the soap to gain a rate for its generation. Still, for all analyses performed after the fretting experiment, we cannot provide data about the development of tribomaterial over time. According to Table 11 and Table 12, as well as the Cr/Co and Cr/Fe ratios within the tribomaterial, we choose Ti and Cr as the main chemical elements to form reaction products for the tribomaterial. This follows the models of tribo-oxidation with a passive film growing under tribological loading and spalling off at a certain critical thickness to form a granular wear particle. This model also includes those wear particles that are generated by microcutting and immediately oxidize. Thus, we might underestimate the influence of Mo that, besides being dissolved within the passive layers and particles, further adds to the organic fraction of the tribomaterial by its reaction with albumin [33,35,77,78]. Under such limitations, it can be seen from Table 14 for AHNS/Ti alloy and ZTA/AHNS couples that the contributions from the cylinder materials to the tribomaterial are always larger than that from the pins and are most prominent for Ti6Al4V. 

This is also supported by our earlier findings with other metal/metal and ceramic/metal couples [18,34,38]. From the cylinder side, the contribution of Cr to the tribomaterial is smaller, as it is in general also from the pin side for all couples. While, for the AHNS/Co alloy couples, we cannot tell the source of Cr, it is surprising that the Co alloy/AHNS and AHNS/AHNS couples render the smallest values. There is no correlation between the tribomaterial generation rate and the accumulated frictional work per wear path. This can be simply explained by the fact that the entire SPD layer dissipates the frictional work, while the ICP-MS results stem only from that fraction which contains organic constituents. Still, this also gives rise to the assumption that, even though the most obvious wear appearances like scratches point towards mechanical-dominated wear submechanisms, there should be something else being characteristic for this tribological behavior under ultra-mild fretting corrosion. By the absence of any signs of surface fatigue (e.g., indentations, delaminations, and microcracks) as well as of adhesion (e.g., materials transfer), the influence of tribocorrosion and tribo-oxidation visible by the OCP should be discussed. In liquid media, tribocorrosion is understood as the accelerated release of metal ions from surfaces, resulting from the destruction of a passive film, e.g., during microploughing or microcutting [32]. Tribo-oxidation is known for an extreme acceleration of the generation of oxide layers on tribologically loaded surfaces. In liquid media, we can understand tribo-oxidation as the mechanism that repassivates surfaces. We showed already that, in a CoCrMo/TiAlV couple, the SPD layer tribocorrodes away extremely fast on the Co side, while such a layer in other tribosystems can act like a passive film in electrochemical polarization experiments [31,79]. Nevertheless, the immediate repassivation kinetics of such a tribologically loaded SPD layer has been investigated, showing that, in proteinaceous media, repassivation is slowed down [80].

But, if—besides the release of wear particles—microcutting, microploughing, and mechanical mixing results in the release of metal ions that can react with the elements and constituents of the lubricant, the ΔOCP might render some information. Certainly, a problem arises from the fact that, with metal/metal couples, we do not know the exact source and distribution of the released ions. Thus, we start with ceramic/metal couples, assuming the release of ions is limited to the metal side of the contact from the ridges (Table 15). It immediately becomes obvious by the ΔOCP that most ions are released from the Ti side, while the Co alloy releases less and the AHNS releases the least. But this picture changes for the metal/metal couples (Table 15). Now, besides the Ti, the Co, Cr, and Mn ions are also released, and the ΔOCP are about the same for all couples and similar to the ZTA/Co alloy.

Now, the ΔOCP does not depend on tribocorrosion alone, since the OCP after run-in falls into a relatively constant plateau, which is governed by the balance between the depassivating (tribocorrosion) and repassivating (tribo-oxidation) processes.

**Figure 22:**
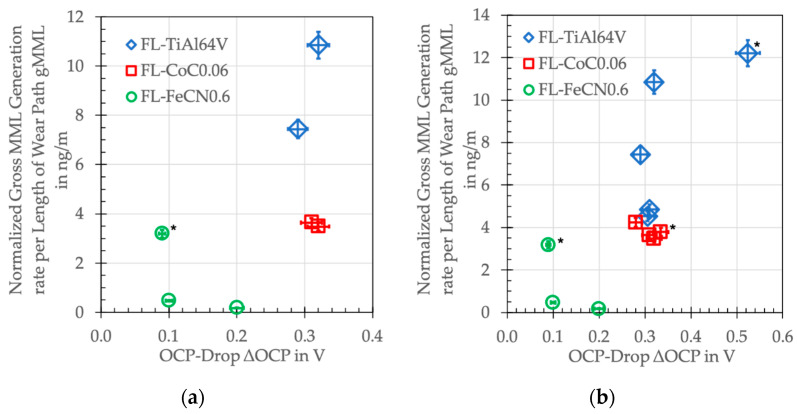
Normalized gross MML generation rate g_MML_ in ng/m and the ΔOCP in V of the different combinations of materials (* ZTA/metal couples). (**a**) This work; (**b**) This and former work [20,39,44].

With all the uncertainties coming from our approach, it becomes clear that there is a tendency of the balance between tribocorrosion and tribo-oxidation represented by the ΔOCP being related to the MML generation rate (Figure 22). Obviously, Ti ions contribute most (Table 12), while, as soon as only Cr ions are around, the processes are slowed down. In this rough approach, and within the limits of the resolution of the ICP-MS and our normalization procedure, AHNSs appear to release the least ions as to the ΔOCP, and as a result, g_MML_ reaches the smallest values. Since any underlying elementary chemical processes triggered by ions, particles, as well as their interactions and their kinetics remain unknown, we cannot discuss this any further. Nevertheless, it becomes obvious that the release of ions from the surfaces in contact has a marked influence on the generation rate of the mechanically mixed layer by chemical reactions with the organic and inorganic constituents of the lubricant.

With respect to clinical applications, it might appear beneficial if fewer metal ions are released into the biological environment. But it is certainly questionable whether the related slower generation of the MML, which is needed for its boundary-lubricating capabilities, also would lead to a better tribological behavior.

### 4.5. The Normalized Gross Wear Rate under Fretting Corrosion w_FC_

The metal ion concentrations per wear path within the serum, as shown in Table 16, represent the matter that completely left the tribosystem in the form of ions and particles from both bodies and, therefore, are used to describe the gross material loss rate. In order to gain a wear rate, and to compare it to former experiments, we calculated the normalized wear rate w_FC_, as described in Section 2.2.4. Thus, wear rates cannot be given for the ZTA ceramic, but only for the metal counterbodies. Table 16 gives an overview of the couples in this paper showing very small wear rates within the ultra-mild range of ng/m.

Some of the numbers in Table 13 are at the verge of the resolution of the used method, and we will only rank them and not give any full quantification. From this, we can read that the pins wear less than the cylinders, except for the AHNS/Ti alloy couples. This is in line with earlier findings that the Ti alloy mainly generates grainy tribomaterial (compare Table 12 and Table 13), which can act as an abrading agent on the counterface [33]. We used this effect to explain the so-called imprinting failure appearances of the head–taper connections that were reported in retrieval studies [81]. We have shown that the main wear mechanism is tribocorrosion on the counterface, triggered by the wear products generated on the Ti side [31]. Thus, we conclude that Ti mainly contributes to the tribomaterial and very little to the wear loss, while, for Co, it is exactly the opposite (compare Table 12 and Table 13). With Mn, the metal ion concentration of the soap is always smaller than that of the serum, but the numbers are always an order of magnitude smaller compared to Ti in the soap and Co in the serum. This is partly related to the fact that Mn is the alloying element and not the base element. Additionally, there are only very few Mn-based tribochemical reaction products within the tribomaterial. With Cr, an alloying element within the Co alloy and the AHNS, it appears to be the other way around, but with always larger differences between the soap and serum compared to Mn. Additionally, from the ZTA/FeCN0.6 couples with about 30 ppb Cr within the soap and about 5 ppb within the serum, we summarized that—like Ti—Cr mainly contributes to the tribomaterial and not to the wear loss.

With a Co alloy cylinder for the AHNS/Co alloy as well as the Co alloy/AHNS couples, the gross wear rates of the pins and cylinders range between 2 and 2.4 ng/m and are larger than those of the AHNS/Ti alloy couples. This is mainly related to the high Co content within the serum and should be mainly based on the tribocorrosion on the Co side [31]. Without the Co alloy bodies, the gross wear rates become smaller, with 1.3 ng/m for the AHNS/AHNS couple and about 0.6 for the AHNS/Ti alloy ones. Thus, the AHNSs appear to show a better behavior than the Co alloy if the submechanisms of the tribochemical reactions, like tribocorrosion and tribo-oxidation, markedly influence the wear behavior. Already in an earlier investigation under more inflammatory conditions, we saw the advantage of FeCN0.9 against CoC0.03 for its better tribochemical properties [54]. This is also indicated by the smaller ΔOCP of the ZTA/AHNS and AHNS/AHNS couples (Figure 4).

**Figure 23:**
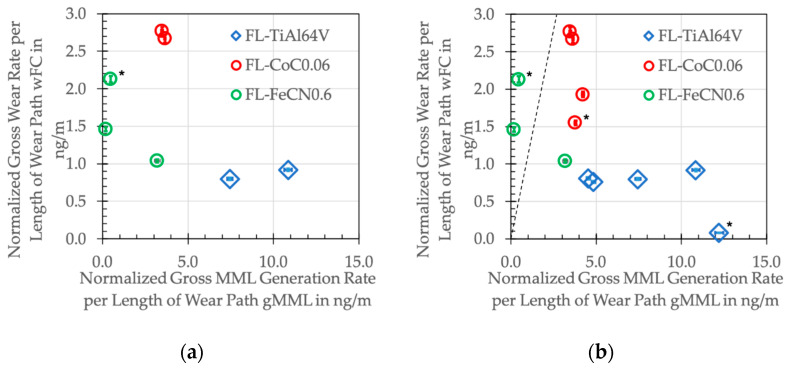
Normalized gross wear rate w_FC_ vs. the normalized gross MML generate rate g_MML_ of the different combinations of materials (* ZTA/metal couples). (**a**) This work; (**b**) This and former work [20,39,44].

Interestingly, all Ti6Al4V couples showed the smallest w_FC_ (Table 16), which gives rise to the question of whether this is somehow related to the largest g_MML_. As can be seen in Figure 23a,b, all the w_FC_ of the fluted Ti6Al4V couples stayed below 1 ng/m, while the MML generation rate could be about 5 to 12 times larger. This would resemble the classical third-bodies approach, in which more third bodies are generated than ejected, underlining their protective nature [19,21,36]. But, as can be seen, there is no direct relation between these parameters. This is also true for all fluted CoC0.06 couples with a g_MML_ of about 4 ng/m but with a w_FC_ ranging from 1.5 to 3 ng/m.

The dashed line in Figure 23b represents the ratio of g_MML_/w_FC_ = 1, elucidating that, for fluted FeCN0.6, the generation rate of the MML is smaller than that of the wear rate, which appears to be impossible. How can there be less generated than ejected? In order to explain this, one has to keep in mind that we only measure the rates on the basis of ions from the inorganic constituents of the solid bodies of this tribosystem. Thus, we have no information about the fraction generated from the BCS. We know from the EDS and RS analyses (Table 11) that there must be quite a high amount of the organics, as well as their reaction products (the high amount of C that cannot come from the alloys), but it is impossible to quantify the amount.

Still, the results contain some information about the importance of this nanostructured multiphase organic–ceramic–metallic composite in order to allow for ultra-mild wear rates. For this, we should point towards two competing aspects of liquid and solid tribochemical reaction products within the interfacial medium in such boundary-lubricated contact situations. Firstly, we found the remains of the liquid phase, as well as other reaction products, by EDS and RS within the scratched grooves of both bodies. Thus, the remains of the liquid constituents represent a substantial part of the tribomaterial and their reaction products being native, denatured and cleaved proteins, sp2-hybridized C, and oxidic tribochemical reaction products. The fact that we found this within the scratched wear grooves on both bodies shows that they have not been ejected from the system and acted as boundary lubricants. Secondly, the grainy debris was mostly found in the form of piles which were pushed out of the contact areas. Thus, after its generation, it should carry the load like a solid lubricant. In the course of loading, it will then move within the real contact spots but not pile up or agglomerate into larger particles, which would give rise to submechanisms of abrasion, surface fatigue, and adhesion. From experiments with fine-machined and fluted cylinders, we know that the dwell time within the contact areas of such grainy tribomaterial is quite important [38]. With a fine-machined cylinder topography, the particles agglomerate and lead to a larger wear loss [18,33]. With fluted ones, the grainy debris is generated, carries the load, and leaves the real contact areas directly in the direction of the relative velocity [34,38]. This combination of solid–liquid boundary lubrication with parts remaining in the contact and parts leaving it allows for such ultra-mild wear rates, while their steady delivery is ensured by the submechanisms of abrasion, like microploughing and microcutting, as well as of tribochemical reactions, like tribocorrosion, tribo-oxidation, and mechanical mixing.

In combination with fluted Ti6Al4V, there is no difference in the gross wear rates of the tribosystems investigated independent of the counter-materials. In general, the w_FC_ of the AHNS-containing couples are in the same order of magnitude of ultra-mild wear as those of CoC0.06. Thus, AHNSs appear neither better nor worse under gross slip fretting corrosion and might, therefore, present a Co- and Ni-free alternative for possible biomedical applications.

### 4.6. Implications

For any clinical application, we have to consider that there are other and possibly more relevant failure mechanisms for real taper joints than just gross slip fretting corrosion [16,82]. We did show earlier that, e.g., Mn ions do not act as detrimental on bone cells as, e.g., Ni ions [47], but even under ultra-mild wear, metal ions, reaction products, and wear particles are ejected from AHNSs. These will be recognized as foreign bodies and might, therefore, still trigger cellular side-effects.

## 5. Limitations

- The contact stiffness of the test rig differs markedly from the stiffness of a real taper joint.
- The topography chosen does not represent any of today’s clinically applied taper topographies.
- The parameters chosen only mimic the gross slip fretting corrosion of taper junctions and do not cover any other mechanisms leading to reaction products causing trunnionosis.
- The fraction of wear particles within the BCS and, therefore, their contribution to the gross wear loss measured by ICP is not known.
- The ICP-MS results are at the verge of the resolution (≈1 ppb) of the substances for the current protocol.
- The analyses used did not allow for a full quantification of all possible—especially organic—boundary lubricants generated by the tribosystem.

## 6. Conclusions and Outlook

Gross slip fretting corrosion experiments of austenitic high-nitrogen steels against different counterbodies in BCS at 37 °C brought about the following results:

- The tribological behavior is characterized by the acting submechanisms of abrasion (microploughing and microcutting) and of tribochemical reactions (tribocorrosion, tribo-oxidation, and mechanical mixing).
- Certain constituents of the in situ generated tribomaterial (third bodies) allow for ultra-mild fretting wear rates for all investigated combinations of bodies and counterbodies.
- The lowest gross wear rates were found by a combination of both austenitic high-nitrogen steels investigated against fluted Ti6Al4V.
- As published before, such steels could be a Co- and Ni-free alternative to CoCrMo alloys.

Future work should contain the following aspects:

- For the ultra-mild wear and the resolution of the ICP (≈ 1 ppb), future tests should run much longer up to a minimum of 10^6^ cycles.
- Further research will be necessary in order to understand the immediate in situ repassivation reactions and kinetics of severely plastically deformed tribomaterial.
- The wear particles should be characterized as well as the cell reactions they might initiate and/or promote.

## Figures and Tables

**Table 1:** Chemical composition and hardness HV10 of the materials tested.

Weight %											ISO	HV10
ZTA (acc. to EDS)								Al	Zr	O		
								37	17	39	6474-2	1732 ± 6
	C	N	Co	Mn	Cr	Mo	Ti	Al	V	Fe		
CoC0.06	0.06	-	Bal	-	27.6	5.7	-	-	-	0.19	5832-12	400 ± 11
FeCN0.9	0.08	0.88	-	14.0	18.0	3.5	-	-	-	Bal	~5832-9 (1.4452)	260 ± 5
FeCN0.6	0.17	0.56	-	12.3	17.2	2.9	-	-	-	Bal	5832-9 (1.3808)	327 ± 12
Ti6Al4V	0.015	-	-	-	<0.01	<0.01	Bal	6.0	3.88	0.13	5832-3	296 ± 2

**Table 2:** Roughness values for the specimens tested in µm.

Type	Material	R_a_ in µm
Polished Pin	FeCN0.9	0.003 ± 0.001
	FeCN0.6	0.003 ± 0.001
	CoC0.06	0.007 ± 0.001
	ZTA	0.017 ± 0.002
Fluted Cylinder	Ti6Al4V	2.38 ± 0.12
	CoC0.06	5.63 ± 0.29
	FeCN0.6	7.65 ± 0.33

**Table 3:** Combinations of the pins and cylinders tested.

		Pins			
	Materials	FeCN0.9	FeCN0.6	CoC0.06	ZTA
Fluted Cylinders	Ti6Al4V	This work	This work	[38]	[34]
	CoC0.06	This work	This work	[34]	[34]
	FeCN0.6	This work	-	This work	This work

**Table 4:** Criteria A, B, and D of the fretting experiments analyzed according to [56].

		Values Averaged Over All Cycles of All Tests						
Body (Cylinder)		FL-Ti6Al4V		FL-CoC0.06		FL-FeCN0.6		
Counterbodies (Pins)		FeCN0.9	FeCN0.6	FeCN0.9	FeCN0.6	FeCN0.9	CoC0.06	ZTA
A_t_ > 0.2	Work Ratio A	0.68 ± 0.05	0.71 ± 0.01	0.72 ± 0.02	0.65 ± 0.09	0.66 ± 0.06	0.75 ± 0.01	0.89 ± 0.01
D_t_ > 0.26	Sliding Ratio D	0.76 ± 0.07	0.79 ± 0.03	0.72 ± 0.01	0.63 ± 0.07	0.71 ± 0.06	0.79 ± 0.02	0.91 ± 0.01
B_t_ > 0.77	System-Free Parameter B	0.91 ± 0.08	0.91 ± 0.04	1.0 ± 0.03	1.07 ± 0.1	0.97 ± 0.09	0.95 ± 0.02	0.91 ± 0.01

**Table 5:** Mean values and standard deviations of the mean frictional work per cycle W_d/c_ and the accumulated frictional work over all cycles W_acc_ of all runs.

Pin/Fluted Cylinder		W_d/c_ in mNm	W_acc_ in Nm
AHNS/Ti alloy	FeCN0.9/FL-Ti6Al4V	1.13 ± 0.04	45.3 ± 1.7
	FeCN0.6/FL-Ti6Al4V	1.08 ± 0.07	43.2 ± 2.9
AHNS/Co alloy	FeCN0.9/FL-CoC0.06	1.33 ± 0.14	57.1 ± 1.8
	FeCN0.6/FL-CoC0.06	1.35 ± 0.06	53.1 ± 5.6
Co alloy/AHNS	CoC0.06/FL-FeCN0.6	1.48 ± 0.07	59.1 ± 2.8
AHNS/AHNS	FeCN0.9/FL-FeCN0.6	1.24 ± 0.15	49.6 ± 6.0
ZTA/AHNS	ZTA/FL-FeCN0.6	0.92 ± 0.09	37.0 ± 3.7

**Table 6:** Mean ΔOCP between the start and steady state.

Pin/Fluted Cylinder		ΔOCP in V
AHNS/Ti alloy	FeCN0.9/FL-Ti6Al4V	0.29 ± 0.04
	FeCN0.6/FL-Ti6Al4V	0.32 ± 0.05
AHNS/Co alloy	FeCN0.9/FL-CoC0.06	0.32 ± 0.01
	FeCN0.6/FL-CoC0.06	0.31 ± 0.04
Co alloy/AHNS	CoC0.0.6/FL-FeCN0.6	0.10 ± 0.02
AHNS/AHNS	FeCN0.9/FL-FeCN0.6	0.20 ± 0.09
ZTA/AHNS	ZTA/FL-FeCN0.6	0.09 ± 0.02

**Table 7:** EDS–spot analyses within and between the wear grooves.

Element	Within Wear Grooves		Grainy Debris between Wear Grooves			
C	11.58	18.89	39.19	46.22	39.28	51.14
O			10.06	9.71	28.68	17.97
P			0.52	0.51	0.64	0.73
S			1.46	1.33	1.54	1.70
Ca			0.15	0.17	0.27	0.25
Cr	25.08	23.18	15.43	10.93	9.79	6.56
Mn	0.64			0.29		
Fe			0.66	0.97	0.70	1.12
Co	56.82	52.45	23.90	18.33	6.08	5.60
Mo	5.11	4.94				

**Table 8:** EDS–spot analyses of white particles within debris on the cylinder side.

Element	Debris Particles		
C	44.05	42.05	51.95
O	26.07	22.24	20.05
P	1.60	1.41	1.56
S	1.32	1.64	1.99
Ca	0.47	0.42	0.53
Cr	13.24	10.98	12.19
Mn	1.05	0.93	1.26
Fe	11.13	9.04	10.33

**Table 9:** EDS–spot analyses of white particles within debris on the pin side.

Element	Debris Particles						
C	56.56	50.01	51.89	47.55	56.03	56.88	45.12
O	20.53	23.85	18.75	24.68	22.57	23.36	27.48
Si	0.22	0.10	0.28		0.20	0.16	0.06
P	1.10	0.55	0.56	0.66	1.03	1.27	0.52
S	2.55	1.51	1.74	1.49	1.84	1.80	0.81
Ca	0.31	0.16	0.17	0.25	0.35	0.42	0.14
Cr	9.44	5.00	9.18	6.18	7.90	8.24	2.77
Mn	1.21	0.61	2.73	0.57	1.08	0.80	0.32
Fe	8.08	3.56	14.70	3.35	9.01	7.08	3.28

**Table 10:** EDS–spot analyses on top of the ridges and within the valleys.

Element	Ridge	Debris		
C	8.54	52.36	53.35	21.05
O	2.45	16.76	15.92	4.85
Al		0.09		
Si	0.44	0.17	0.15	0.38
P		0.55	0.60	0.24
Ca		0.28	0.43	
Cr	16.99	6.37	5.92	12.74
Mn	11.16	3.52	3.29	8.56
Fe	57.61	19.91	17.94	46.12

**Table 11:** Inorganic and organic constituents of the reaction layers on the surfaces before and after fretting experiments after sonication in ethanol.

	AHNS FeCN0.9	BCS
Before the fretting experiment	MoO_3_, Cr_3_O_8_	tryptophan, cysteine, phenylanaline, lipids, tyrosine, fatty acids, native albumin, globulin
After the fretting experiment within the immediate contact area	+FeCr_2_O_4_, CrO_4_^2−^, CrO_3_, Cr_2_O_5_, MnMoO_4_	+denatured, cleaved proteins, sp^2^-hybridized C
	+TiO_2_, Cr_2_O_3_, MoO_2_, Fe_2_O_3_, Mn^3+^-OOH	
After the fretting experiment materials pushed out of the immediate contact area	all of the above	

**Table 12:** Metal ion concentration in ppb within soap (contributions from the cylinder are marked in green and those from the pins are marked in blue for better clarity).

Pin/Cylinder		Soap			
		Cr	Mn	Co	Ti
AHNS/Ti alloy	FeCN0.9/FL-Ti6Al4V	11.9 ± 0.04	11.5 ± 0.4	-	460 ± 130
	FeCN0.6/FL-Ti6Al4V	34.6 ± 9.1	12.2 ± 8	-	650 ± 50
AHNS/Co alloy	FeCN0.6/FL-CoC0.06	40.2 ± 3.8	9.4 ± 0.9	38.6 ± 10.8	-
	FeCN0.9/FL-CoC0.06	42.1 ± 8.6	11.3 ± 0.4	32.4 ± 0.2	-
Co alloy/AHNS	CoC0.06/FL-FeCN0.6	44.9 ± 1.3	18.2 ± 1.3	41.1 ± 8.2	-
AHNS/AHNS	FeCN0.9/FL-FeCN0.6	16.0 ± 3.9	11.9 ± 3.4	-	-
ZTA/AHNS	ZTA/FL-FeCN0.6	30.1 ± 9.5	18.1 ± 5.9	-	-

**Table 13:** Metal ion concentration within serum (contributions from cylinders are shown in green and those from pins in blue if the system allows a determination).

Pin/Cylinder		Serum			
		Cr	Mn	Co	Ti
AHNS/Ti alloy	FeCN0.9/FL-Ti6Al4V	9.2 ± 1.9	17.6 ± 2.1	-	3.0 ± 1.7
	FeCN0.6/FL-Ti6Al4V	12.3 ± 4	19.6 ± 5.0	-	3.0 ± 0.6
AHNS/Co alloy	FeCN0.9/FL-CoC0.06	31.9 ± 4.2	13.6 ± 0.1	252 ± 4.5	-
	FeCN0.6/FL-CoC0.06	16.8 ± 2.9	22.1 ± 5.3	230 ± 5.8	-
Co alloy/AHNS	CoC0.06/FL-FeCN0.6	7.9 ± 0.6	37.4 ± 1.7	86.7 ± 9.2	-
AHNS/AHNS	FeCN0.9/FL-FeCN0.6	6.9 ± 0.4	35.1 ± 4.7	-	-
ZTA/AHNS	ZTA/FL-FeCN0.6	4.7 ± 0.4	25.4 ± 7.2	-	-

**Table 14:** The accumulated frictional work per wear path W_acc/l_ and the normalized gross generation rates of the mechanically mixed layers g_MML_ in ng/m.

	Polished Pin	Fluted Cylinder	W_acc/l_ in mNm/m	g_MML_ in ng/m
AHNS/Ti alloy	FeCN0.9	Ti6Al4V	573 ± 20	7.4
	FeCN0.6	Ti6Al4V	537 ± 33	10.9
AHNS/Co alloy	FeCN0.9	CoC0.06	749 ± 35	3.5
	FeCN0.6	CoC0.06	692 ± 72	3.6
Co alloy/AHNS	CoC0.06	FeCN0.6	759 ± 31	0.5
AHNS/AHNS	FeCN0.9	FeCN0.6	655 ± 86	0.2
ZTA/AHNS	ZTA *	FeCN0.6	472 ± 44	3.2 *

* ICP analyses of Al released from ZTA could not be performed due to the high amount of Al carried over from NBCS.

**Table 15:** Mean ΔOCP between the start and steady state for ZTA/metal couples of this and former work [20,39,44].

Polished Pin/Fluted Cylinder		ΔOCP in V
ZTA/Ti alloy	ZTA/FL-Ti6Al4V	0.52 ± 0.04
ZTA/Co alloy	ZTA/FL-CoC0.06	0.32 ± 0.01
ZTA/AHNS	ZTA/FL-FeCN0.6	0.09 ± 0.02
Co alloy/Ti alloy	CoC0.06/FL-Ti6Al4V	0.31 ± 0.07
AHNS/Ti alloy	FeCN0.9/FL-Ti6Al4V	0.29 ± 0.04
	FeCN0.6/FL-Ti6Al4V	0.32 ± 0.06

**Table 16:** The accumulated frictional work per wear path W_acc/l_ and the normalized gross wear rates w_FC_ in ng/m.

	Polished Pin	Fluted Cylinder	W_acc/l_ in mNm/m	w_FC_ in ng/m
AHNS/Ti alloy	FeCN0.9	Ti6Al4V	573 ± 20	0.80
	FeCN0.6	Ti6Al4V	537 ± 33	0.92
AHNS/Co alloy	FeCN0.9	CoC0.06	749 ± 35	2.77
	FeCN0.6	CoC0.06	692 ± 72	2.67
Co alloy/AHNS	CoC0.06	FeCN0.6	759 ± 31	2.13
AHNS/AHNS	FeCN0.9	FeCN0.6	655 ± 86	1.46
ZTA/AHNS	ZTA *	FeCN0.6	472 ± 44	1.04 *

* ICP analyses of Al released from ZTA could not be performed due to the high amount of Al carried over from NBCS.

## Data Availability

The data presented in this study are available on request from the corresponding author due to this research was carried out and funded in full by private and not public entities.

## Abbreviations

AB	abrasion (wear mechanism)
AD	adhesion (wear mechanism)
APT	atom probe tomography
BCS	phosphate-buffered bovine calf serum solution
BSE	backscattered electron
CWLM	confocal white-light microscopy
EDS	energy-dispersive X-Ray spectroscopy
FL	fluted
ICP-MS	inductively coupled plasma–mass spectroscopy
MML	mechanically mixed layer
NBCS	newborn calf serum
OCP	open corrosion potential
RS	Raman scattering
SF	surface fatigue (wear mechanism)
SEM	scanning electron microscopy
SPD	severe plastic deformation
TCR	tribochemical reactions (wear mechanism)
TEM	transmission electron microscopy
ZTA	zirconia-toughened alumina
